# Cervical Spinal Cord Stimulation for Functional Rehabilitation After Spinal Cord Injury: A Systematic Review of Preclinical and Clinical Studies

**DOI:** 10.3390/life16010179

**Published:** 2026-01-22

**Authors:** Maximilian C. Wankner, Veerle Visser-Vandewalle, Pablo Andrade, Petra Heiden

**Affiliations:** 1 Department of Neurosurgery, Faculty of Medicine and University Hospital Cologne, University of Cologne, 50937 Cologne, Germany; 2 Department of Stereotactic and Functional Neurosurgery, Faculty of Medicine and University Hospital Cologne, University of Cologne, 50937 Cologne, Germany; veerle.visser-vandewalle@uk-koeln.de (V.V.-V.); pablo.andrade-montemayor@uk-koeln.de (P.A.); petra.heiden@uk-koeln.de (P.H.)

**Keywords:** spinal cord injury, cervical SCI, neuromodulation, epidural stimulation, transcutaneous spinal stimulation, upper limb motor recovery, neuroplasticity, neurorehabilitation

## Abstract

Cervical spinal cord injury causes severe functional impairment with limited spontaneous recovery, and while spinal cord stimulation has emerged as a promising neuromodulatory strategy, evidence for cervical applications remains fragmented. To address this gap, we conducted a systematic review synthesizing preclinical and clinical evidence on cervical spinal cord stimulation for functional rehabilitation following spinal cord injury. The review was registered on PROSPERO (CRD420251088804) and conducted in accordance with PRISMA guidelines, with PubMed, Embase, IEEE Xplore, and Web of Science searched from inception to July 2025 for animal and human studies of cervical spinal cord stimulation, including epidural, intraspinal, and transcutaneous approaches, reporting functional neurological outcomes. Risk of bias was assessed using the Cochrane RoB 2 and ROBINS-I tools, and due to substantial heterogeneity, results were synthesized narratively. Thirty-one studies comprising 119 animals and 156 human participants, met inclusion criteria. Across studies, outcome measures such as GRASSP, ISNCSCI, and dynamometry consistently demonstrated improvements in hand strength, dexterity, and voluntary motor activation. Several studies also reported gains in sensory and autonomic function, whereas respiratory outcomes were infrequently assessed. Adjunctive interventions, including cortical stimulation, brain–computer interface priming, and task-specific training frequently augmented recovery. Adverse events were generally mild, although overall risk of bias was predominantly serious. Overall, cervical spinal cord stimulation demonstrates preliminary assistive and therapeutic effects on motor recovery, with additional sensory, autonomic, and potential respiratory benefits.

## 1. Introduction

Spinal cord injury (SCI) affects primarily young and middle-aged adults, with traumatic cases occurring at a mean age of 35 years and individuals aged 21–40 accounting for over 40% of cases [1,2]. Cervical injuries frequently cause tetraparesis or tetraplegia, resulting in loss of independence in daily activities, caregiver reliance, and, in high cervical lesions, respiratory compromise requiring ventilatory support [3]. Chronic sequelae, including spasticity, neuropathic pain, cardiovascular dysregulation, and pressure ulcers, contribute further to long-term morbidity [4].

The intrinsic potential for recovery after high cervical SCI remains limited. Neurological improvement generally plateaus within the first year, and gains are modest even under intensive rehabilitation [5,6,7,8]. While incremental improvements can meaningfully impact independence, substantial restoration of function is rare in motor-complete or near-complete cervical SCI. Current treatment approaches focus on acute surgical decompression of the spinal cord and stabilization, prevention of secondary injury, pharmacological management, and long-term multidisciplinary rehabilitation. Although advances in rehabilitation techniques have led to incremental improvements, they remain insufficient for restoring meaningful function in patients with severe cervical SCI. Crucially, traditional therapies are limited by the absence of interventions that can directly modulate spinal circuitry and promote recovery beyond the modest natural course.

Spinal cord stimulation (SCS) has emerged as a promising neuromodulation strategy to address this gap. Originally introduced for analgesia in the 1960s based on the gate control theory of pain [9,10], subsequent work showed that activating spinal networks can also influence motor output. In individuals with chronic motor-complete paraplegia, lumbosacral epidural stimulation combined with intensive rehabilitation has enabled weight-bearing standing, volitional control of paralyzed muscles, and even overground walking [11,12,13]. These landmark studies established that residual spinal circuits below the lesion can generate functional motor patterns when appropriately facilitated [14].

Following successful demonstrations of SCS at the lumbosacral cord, attention shifted to the cervical spinal cord, given its critical role in upper limb and respiratory function. Several stimulation modalities have been explored, including intraspinal microstimulation (ISMS) via penetrating microelectrodes, epidural electrical stimulation (EES) using dorsal column arrays, and non-invasive transcutaneous stimulation (tSCS) over the dorsal cervical spine [15,16,17,18,19,20,21,22,23,24,25,26]. ISMS offers high anatomical and functional specificity in preclinical models but relies on penetrating electrodes that currently limit near-term clinical translation; EES enables more targeted segmental recruitment than tSCS but requires invasive surgical implantation; and tSCS provides a non-invasive and readily deployable approach at the expense of reduced spatial specificity. Preclinical studies demonstrate that cervical stimulation can selectively recruit forelimb and respiratory circuits and support recovery of skilled motor behaviors after injury [15,16,19,21,27,28,29,30,31,32,33,34,35,36,37,38]. These findings suggest that cervical neuromodulation may provide a means to restore upper limb and respiratory function beyond the modest natural course of recovery.

Against this background, the present systematic review synthesizes preclinical and clinical evidence on cervical SCS for functional rehabilitation after SCI, with a focus on motor, sensory, respiratory, and autonomic outcomes, and evaluates the methodological quality of the available studies.

## 2. Materials and Methods

### 2.1. Search Strategy

This systematic review was conducted and reported in accordance with the Preferred Reporting Items for Systematic Reviews and Meta-Analyses (PRISMA) 2020 guidelines. The review protocol was prospectively registered in PROSPERO (CRD420251088804). Reporting was conducted in accordance with the PRISMA guidelines [39]. The PRISMA 2020 flow diagram was generated using the PRISMA2020 R package and Shiny app version 1.1.1 [40]. A comprehensive search of the published literature was performed in MEDLINE/PubMed, Embase, Web of Science, and IEEE Xplore electronic databases in June–July 2025 by two independent reviewers (M.W. and P.A.), working independently and blinded to each other’s selections to ensure reliability. The systematic search combined both keywords and, where applicable, Medical Subject Headings (MeSH), using the following Boolean string: (“cervical spinal cord stimulation” OR “cervical spinal cord” OR “cervical stimulation”) AND (“spinal cord injury” OR “SCI”) AND (“stimulation” OR “neuromodulation”) AND (“recovery” OR “rehabilitation” OR “function” OR “movement” OR “sensory” OR “motor” OR “autonomic” OR “respiration”). Additionally, the reference lists of all included studies and relevant reviews were manually screened to identify any additional eligible reports not captured by the electronic search.

### 2.2. Study Selection Criteria

Study selection was guided by predefined PICOS (Population, Intervention, Comparison, Outcome, and Study design) criteria [41]. Studies were eligible for inclusion if they met the following criteria: (1) enrolled humans or animals with spinal cord injury; (2) applied electrical stimulation to the cervical spinal cord; (3) reported outcomes in at least one functional neurological domain (motor, sensory, autonomic, respiratory, or related function); and (4) employed an eligible study design, including case series, case reports, cohort studies, conference abstracts, preprints, or randomized controlled trials (RCTs). Studies were excluded if they (1) focused primarily on chronic pain, spasticity, or other non-functional outcomes; (2) targeted stimulation sites outside the cervical spinal cord and its associated roots; (3) involved participants with peripheral nerve injuries; (4) lacked sufficient extractable data, such as reviews, editorials, or protocol descriptions; or (5) were published in languages other than English.

### 2.3. Data Extraction

Two independent reviewers (M.W. and P.A.) screened abstracts, titles, and full texts of results yielded by the search strategy. Standardized data were extracted from eligible studies according to the following metrics: first author’s name and year of publication, type of animal in case of non-human studies, age and gender, clinical characteristics (level of injury, time since injury, ASIA Impairment Scale (AIS) grade), stimulator characteristics (type of device/manufacturer, number of leads, location of leads, and method of lead placement), stimulation parameters (frequency, pulse width, amplitude, stimulation time length, and optimization), functional neurological outcomes, and adverse effects.

### 2.4. Analysis of Neurological Functional Outcomes

Studies reporting functional neurological outcomes were reviewed in detail to identify unique cases and cohorts. Potentially overlapping participants were screened by cross-checking published subject identifiers or explicit references to previously reported cases. When multiple publications described the same cohort, only the most recent or comprehensive report was included. Functional outcomes were synthesized qualitatively across motor, sensory, respiratory, and autonomic domains. Where possible, standardized assessments such as Graded and Redefined Assessment of Strength, Sensibility, and Prehension (GRASSP), ISNCSCI motor scores, and dynamometry were compared descriptively to identify consistent patterns of improvement [42,43]. Due to heterogeneity in study designs, outcome measures, and reporting formats no quantitative pooling or effect size estimation was performed. Instead, findings were narratively summarized to highlight the range and frequency of reported improvements and the contexts in which stimulation was most effective. Functional neurological outcomes in preclinical studies were not systematically analyzed using standardized metrics, as animal experiments employed highly heterogeneous, task-specific outcome measures with incomplete and inconsistent reporting across studies.

To contextualize the magnitude of reported changes, frequently used clinical outcome scales are briefly summarized here. The ISNCSCI Upper Extremity Motor Score (UEMS) ranges from 0 to 50, with higher scores indicating better motor strength across ten key muscles in the upper limbs [43]. GRASSP is reported as separate subscores (not a single total): Strength, 0–50 per limb; Sensibility, 0–12 per limb; Prehension Ability, 0–30 per limb; and Prehension Performance, which is time-based with no maximum score [42]. Neurological severity was categorized using the ASIA Impairment Scale (AIS), where AIS A denotes motor and sensory complete injury and AIS E denotes normal function [44]. Functional independence was commonly measured using the Spinal Cord Independence Measure III (SCIM-III), which ranges from 0 to 100 and captures self-care, respiration, sphincter management, and mobility [45].

### 2.5. Bias Assessment

Two independent reviewers (M.W. and P.A.) assessed the risk of bias for all included human studies. Version 2 of the Cochrane risk-of-bias tool for randomized trials (Rob-2) was applied to randomized clinical trials and the ROBINS-I tool was used for non-randomized studies [46,47]. Risk of bias for preclinical animal studies was not formally tabulated but common limitations, including small sample sizes, heterogeneous injury models, and absence of blinding, are addressed in the Discussion.

## 3. Results

### 3.1. Study Selection

**Figure 1:**
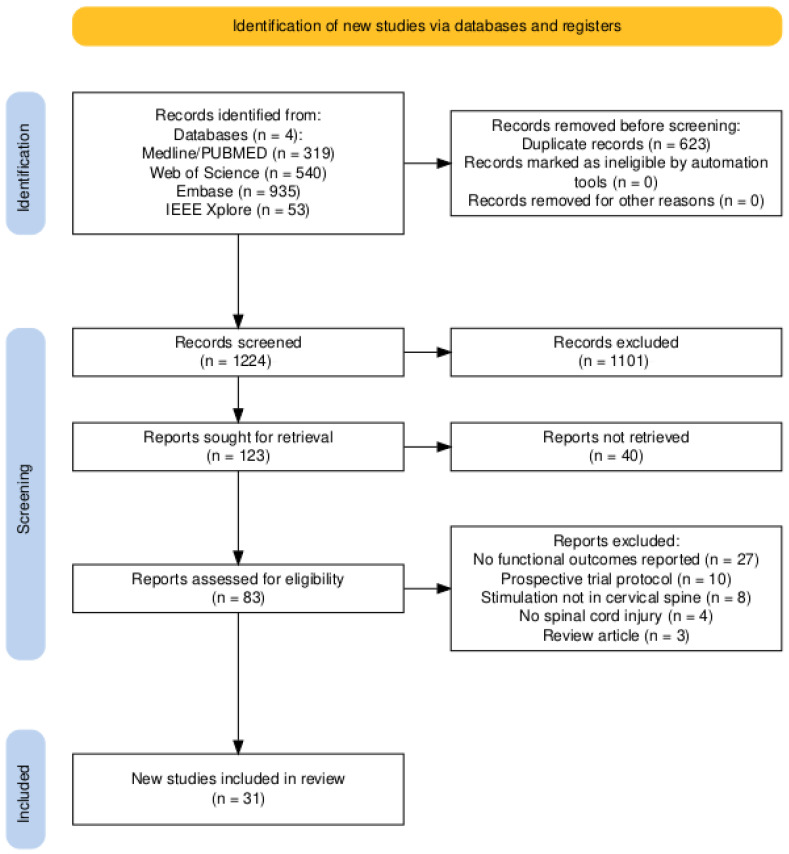
PRISMA flow diagram describing the selection process.

The database search identified 1847 records from PubMed/Medline, Web of Science, Embase, and IEEE Xplore. After removal of 623 duplicate records, 1224 records were screened by title and abstract. Of these, 1101 records were excluded during initial screening. A total of 123 reports were sought for retrieval, of which 40 reports were not retrieved. Eighty-three full-text reports were assessed for eligibility. Fifty-two reports were excluded for the following reasons: no functional outcomes reported (n = 27), prospective trial protocols (n = 10), stimulation not targeting the cervical spine (n = 8), absence of spinal cord injury (n = 4), or review articles (n = 3). In total, 31 studies met the inclusion criteria and were included in the qualitative synthesis. Figure 1 presents the PRISMA 2020 flow diagram summarizing the study selection process [39].

### 3.2. Study and Patient Characteristics

The included 31 studies were published between 2013 and 2025 and involved a total of 275 participants (156 humans, 119 animals) with cervical SCI. Study designs included five case reports, ten prospective case series, three RCTs, one conference abstract, one preprint, and eleven preclinical animal studies. Each study focused on only one type of interface: ISMS was investigated in two, tSCS in twenty-one, and EES in eight studies. Only one study investigated EES in humans with SCI. Injury chronicity varied from acute (e.g., same day) to 32 years post injury, and neurological severity ranged from AIS A–D, where applicable. Where follow-up assessments were carried out, duration ranged from 2 weeks to 9 months. Table 1 summarizes key study and patient characteristics.

### 3.3. Stimulation Location and Parameters

Stimulation parameters varied considerably across both animal and human studies (Table 2). Reported frequencies ranged from 20 to 300 Hz, with pulse widths typically between 200 and 1000 µs and amplitudes titrated to elicit visible or EMG-detected motor responses without discomfort or tissue injury. Electrode placement and optimization strategies differed substantially among stimulation modalities. ISMS was applied in preclinical rodent and primate studies using penetrating microelectrodes inserted directly into the ventral horn, most often from C6 to C7 or T1. These sites reliably recruited forelimb and hand muscles, with short pulse widths (200–300 µs) and variable frequencies and currents. Optimization involved systematic mapping of motor pools to identify electrode sites producing selective grasping or reaching movements. EES in animal models employed electrodes placed over the dorsal surface of the cervical enlargement, frequently lateralized to the dorsal root entry zones to maximize segmental recruitment. Frequencies typically ranged from 20 to 100 Hz with pulse widths between 200 and 500 µs. A respiratory-focused study in rats positioned electrodes at C3–C5 to engage phrenic motoneurons and elicit inspiratory activity. In human studies, stimulation optimization was guided by intraoperative neurophysiological monitoring or iterative adjustment to maximize functional responses. Transcutaneous spinal cord stimulation studies placed large surface electrodes over the posterior neck, typically spanning C3–C5, with reference electrodes over the iliac crests or shoulders. Later work often employed two adjacent cervical electrodes or three-patch configurations to broaden current spread. Frequencies ranged from 20 to 50 Hz, sometimes combined with carrier frequencies of 5 kHz to reduce discomfort and improve current penetration. Pulse widths varied from 500 to 1000 µs, and amplitudes reached 40–200 mA depending on electrode configuration and participant tolerance. Optimization strategies included titration to evoke surface motor evoked potentials (sMEPs), motor pool mapping, or real-time adjustments based on voluntary motor output during training tasks. Collectively, these findings indicate that ISMS enables selective recruitment of cervical motor pools but remains limited to preclinical applications. EES allows segmental control of forelimb and respiratory circuits yet requires surgical implantation, whereas tSCS provides a non-invasive alternative that produces broader, less specific activation patterns.

### 3.4. Outcomes Measured

Motor recovery was the most consistently assessed outcome domain. The synthesis below primarily reflects standardized outcome reporting in human studies, as functional outcomes in animal experiments were heterogeneous and often reported using non-uniform or task-specific measures. Improvements were captured by standardized tools including GRASSP, ISNCSCI upper extremity motor subscores, and dynamometry, supplemented by patient-reported outcomes and task-based assessments (Table 3).

GRASSP outcomes were most frequently reported for tSCS, assessed in nine studies that consistently demonstrated improvements in strength and prehension subscores. Inanici et al. documented a 52-point increase in total GRASSP score, with persistent benefits for more than three months after stimulation ceased; in their later study, similar improvements were maintained up to six months [48,49]. Zhang et al. reported 10–33-point increases in GRASSP scores, with multi-session stimulation plus training producing sustained gains at one to three months [50,51]. Benavides et al. showed measurable GRASSP improvements even after a single tSCS session, while Capozio et al. reported gains in 4 of 5 patients [52,53]. Additional single-patient or small series further reinforced consistent GRASSP improvements when tSCS was paired with adjunctive interventions such as brain–computer interface (BCI) priming or robotic exoskeleton training [54,55]. Similar trends were observed in the EES study by Lu et al., showing improved voluntary control and task performance in two patients after epidural stimulation [18].

ISNCSCI motor scores also demonstrated consistent improvements. Six tSCS studies reported increases of up to 23 points, often exceeding minimally important difference thresholds [48,49,53,54,56,57]. In a multicenter trial of 60 participants, Moritz et al. found that 72% of participants surpassed this threshold [56]. Some studies noted neurological level improvements in one to two segments, though sensory recovery did not consistently follow dermatome maps. Lu et al. documented sustained session-to-session increases in Upper Extremity Motor Scores in two patients treated with cervical EES [18].

Dynamometry outcomes were reported in thirteen tSCS studies, showing consistent gains in voluntary grip and pinch strength. Increases ranged from the first detectable grip force in previously paralyzed muscles to large magnitude gains such as a 325% increase in grip strength and >1000% increases in exerted force [51,58,59,60]. Huang et al. demonstrated that residual baseline grip was a strong predictor of response, with robust gains only in participants who had minimal pre-stimulation grip force [61]. Lu et al. similarly reported improved grip force in implanted participants [18].

Respiratory outcomes were less frequently evaluated. Gad et al. demonstrated in a chronic tetraplegic patient that cervical tSCS improved voluntary breathing and coughing, with effects persisting after stimulation was turned off [62]. In animal models, Bezdudnaya et al. showed that epidural stimulation at C4 maintained breathing with normal end-tidal ${\mathrm{CO}}_{2}$ and improved blood pressure [37].

Autonomic outcomes showed variable but promising findings. Inanici et al. observed improved bladder function, thermoregulation, and heart rate control following tSCS [48]. Singh et al. found cervical tSCS to be hemodynamically safe and feasible in a study of seven children with SCI [63]. Effects were inconsistent across studies, reflecting small sample sizes. Autonomic outcomes were not assessed in the single human EES study [18].

Patient-reported outcomes were included in five tSCS studies, emphasizing improved independence in hand use and quality of life. Moritz et al. reported significant improvements in SCIM-III scores and patient-reported outcome scores in parallel with objective motor gains [64]. EES patients similarly reported improvements in mobility and self-care alongside functional gains [18].

### 3.5. Combination Interventions

Several studies combined cervical spinal cord stimulation with additional interventions aimed at enhancing neuroplasticity and functional recovery. Four preclinical studies paired motor cortex stimulation with cervical SCS. These studies all demonstrated that concurrent cortical and spinal stimulation produced greater improvements in corticospinal excitability and forelimb motor outcomes than either intervention alone, suggesting synergistic effects on descending motor pathways and spinal circuitry [19,65,66,67].

Multimodal approaches were also explored in human studies. Samejima et al. conducted a human feasibility case study in which motor intention detected by a BCI was used to prime cervical tSCS. The intervention demonstrated safety and short-term improvements in voluntary hand function, supporting the concept that coupling stimulation with brain-driven signals may enhance recovery [68]. Capozio et al. combined cervical tSCS with motor imagery in eight participants. This single-session study showed acute improvements in manual dexterity and cortical excitability, suggesting that cognitive engagement of motor networks may potentiate stimulation effects [69]. García-Alén et al. performed a 15-patient clinical trial combining cervical tSCS with upper limb robotic-assisted rehabilitation. Additive improvements in strength and functional outcomes were seen in robotic exoskeleton training and tSCS compared to exoskeleton-assisted training alone [55].

Several additional studies integrated cervical tSCS with intensive task-specific training. These studies reported more durable functional improvements than stimulation alone, reinforcing the role of activity-dependent plasticity [50,70]. Collectively, these findings suggest that cervical SCS may be most effective not as a stand-alone therapy but when used with neuromodulatory adjuncts.

### 3.6. Methodological Quality

**Figure 2:**
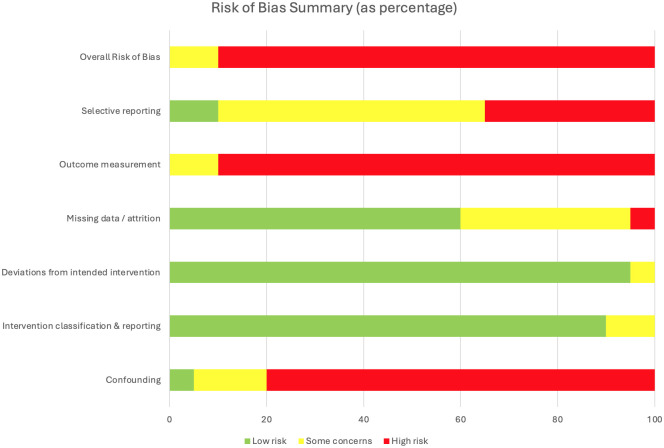
Risk of bias summary for 20 included human studies assessed with the RoB 2 and ROBINS-I tools.

Risk of bias was high across most human studies (Figure 2). Eleven studies were judged to be at serious risk of bias, primarily due to very small sample sizes, heterogeneous injury cohorts, lack of control groups, and unblinded outcome assessments. An additional seven studies were rated as serious–critical, reflecting the limitations of single-patient case reports, exploratory pilot designs, and sparse reporting (including one conference abstract and one preprint), which further constrain generalizability. These two studies were included to reflect the dynamic and rapidly evolving nature of the field; however, as they have not undergone full peer review, their findings should be interpreted with caution. Only two studies achieved lower overall ratings: Huang et al., a sham-controlled crossover trial in 10 participants, was judged as having moderate risk of bias, and the multicenter trial by Moritz et al. (n = 60) was rated moderate due to its larger sample size, standardized protocol, and comprehensive reporting, though it still lacked a sham-control condition [56,61]. Collectively, the evidence base remains dominated by small, uncontrolled studies. Similarly, while preclinical studies provide compelling mechanistic support for cervical spinal cord stimulation, they are constrained by small sample sizes, heterogeneous injury models, and frequent absence of blinding. Together, these limitations underscore that although findings across both animal and human studies are encouraging, the certainty of evidence remains low and highlights the need for larger, rigorously controlled clinical trials. Full results of the human study assessments are summarized in Appendix A, Table A1.

### 3.7. Adverse Effects

Adverse events were infrequently reported. Across 31 studies, no serious complications (e.g., permanent neurological worsening, infection requiring device removal) were documented. Mild or transient effects included skin irritation from transcutaneous electrodes, discomfort or muscle spasms during stimulation, and lead migration in epidural implants. Reporting of safety outcomes was inconsistent, and most studies did not include systematic adverse event monitoring.

**Table 1:** Study characteristics of included animal and human studies.

Authors	Country	Population	Sex	N	Age Range	Injury Level	Injury Duration	Injury Type/Severity	Modality
*Animal studies*									
Kasten et al. (2013) [15]	USA	Rats	F	11	Adult	C4–C5	4 weeks	Contusion injury	ISMS
McPherson et al. (2015) [17]	USA	Rats	F	9	Adult	C4–C5	>8 weeks	Contusion injury	ISMS
Alam et al. (2015) [29]	USA	Rats	F	12	Adult	C4	1–2 weeks	Dorsal funiculi crush	EES
Alam et al. (2017) [30]	USA	Rats	F	5	Adult	C4	1 week	Dorsal funiculi crush	EES
Bezdudnaya et al. (2018) [37]	USA	Rats	M	20	Adult	C1	Acute	Complete transection	EES
Yang et al. (2019) [66]	USA	Rats	F	27	10–12 weeks	C3	11 days	Contusion injury	EES
Samejima et al. (2021) [68]	USA	Rats	F	5	Adult	C4	2 weeks	Contusion injury	EES
Barra et al. (2022) [19]	Switzerland	*Macaca fascicularis*	F	3	3–9 years	C5–C6	1 week	Unilateral CST transection	EES
Pal et al. (2022) [71]	USA	Rats	F	8	Adult	C4	10 days	AAV-based CST inactivation	EES
Song et al. (2016) [67]	USA	Rats	F	6	Adult	C1	1 week	Transection at rostral medualla	tSCS
Zareen et al. (2017) [25]	USA	Rats	F	13	10–12 weeks	C4	2 weeks	Contusion injury	tSCS
*Human studies*									
Lu et al. (2016) [18]	USA	Human	N.R.	2	N.R.	C5–C6	>18 months	AIS B	EES
Murray et al. (2017) [26]	USA	Human	M	1	27 years	C6–C7	9 years	AIS C	tSCS
Inanici et al. (2018) [49]	USA	Human	M	1	62 years	C3	2 years	AIS D	tSCS
Gad et al. (2018) [59]	USA	Human	1F 5M	6	20–62 years	C4–C8	1–21 years	AIS A (n = 0), B (n = 2), C (n = 4), D (n = 0)	tSCS
Benavides et al. (2020) [52]	USA	Human	4F 6M	10	22–64 years	C4–C6	>1 year	AIS A (n = 4), B (n = 3), C (n = 2), D (n = 1)	tSCS
Zhang et al. (2020) [51]	USA	Human	M	1	38 years	C5	15 years	AIS A	tSCS
Gad et al. (2020) [62]	USA	Human	M	1	39 years	C5	9 years	AIS A	tSCS
Tefertiller et al. (2021) [57]	USA	Human	2F 5M	7	18–55 years	C4–C6	15–38 months	AIS A (n = 0), B (n = 4), C (n = 2), D (n = 1)	tSCS
Inanici et al. (2021) [48]	USA	Human	2F 4M	6	28–62 years	C3–C5	1.5–12 years	AIS A (n = 0), B (n = 2), C (n = 2), D (n = 2)	tSCS
Huang et al. (2022) [61]	USA	Human	N.R.	10	22–63 years	C3–C7	2–14 years	AIS A–B	tSCS
McGeady et al. (2022) [54]	UK/Hong Kong	Human	M	1	48 years	C4	12 years	AIS A	tSCS
Zhang et al. (2023) [50]	USA	Human	4M	4	25–78 years	C1–C4	4–9 years	AIS A (n = 1), B (n = 1), C (n = 0), D (n = 2)	tSCS
Oh et al. (2023) [70]	USA	Human	4M	4	23–29 years	C4–C6	2–14 years	AIS A–B	tSCS
García-Alén et al. (2023) [55]	Spain	Human	1F 14M	15	18–70 years	C4–C7	3–10 months	AIS A–D	tSCS
*Human studies*									
Chandrasekaran et al. (2023) [60]	USA	Human	2M	2	20–39 years	C5	4–7 years	AIS A–B	tSCS
Moritz et al. (2024) [56]	International	Human	10F 50M	60	47.2±15.5 years	C2–C7	>1 year	AIS B–D	tSCS
Singh et al. (2024) [63]	USA	Human (Children)	1F 6M	7	6–17 years	C1–C7	>2 years	AIS A (n = 1), B (n = 4), C (n = 1), D (n = 1)	tSCS
Verma et al. (2025) [58]	USA	Human	1F 4M	5	19–67 years	C4–C7	3–18 years	AIS A (n = 3), B (n = 1), C (n = 1), D (n = 0)	tSCS
Capozio et al. (2025) [53]	UK	Human	5F	5	31–65 years	C3–C8	2–30 years	AIS A (n = 0), B (n = 0), C (n = 3), D (n = 2)	tSCS
Capozio et al. (2025) [69]	UK	Human	4F 4M	8	22–72 years	C2–C7	3–32 years	AIS A (n = 0), B (n = 1), C (n = 4), D (n = 3)	tSCS

Abbreviations: AIS = ASIA Impairment Scale; CST = Cortico-Spinal Tract; AAV = Adeno-Associated Virus; N.R. = Not Reported.

**Table 2:** Summary of stimulation parameters and adjunct interventions in animal and human studies.

Authors	Population	Electrode Manufacturer	Electrode Placement	Pulse Width (μs)	Frequency (Hz) (Carrier, kHz)	Amplitude	Adjunctive Intervention	Stimulation Optimization
Intraspinal microstimulation								
Kasten et al. (2013) [15]	Rats	Custom	C6–T1 ventral horn	300	4±1.5	N.R.	–	Set at threshold just to evoke forelimb movement when delivered via a single electrode
McPherson et al. (2015) [17]	Rats	Custom	C6–C7 ventral horn	200	50–100	30–150μA	EMG-synchronized	90% of motor threshold; synchronized ISMS below the injury with volitional motor commands
Epidural stimulation								
Alam et al. (2015) [29]	Rats	Custom	C6 and C8	200	40	400–1000μA	–	70% of motor threshold for mono- and bipolar electrode pairings
Lu et al. (2016) [18]	Human	Boston Scientific	C5–T1	210	2–40	0.1–10 mA	–	Individually adjusted for motor evoked response
Alam et al. (2017) [30]	Rats	Custom	C6 and C8	200	20, 40, 60	N.R.	–	60–70% of sMEP threshold
Bezdudnaya et al. (2018) [37]	Rats	Custom	C3–C5	200–500	100–300	100–500μA	–	Titrated to elicit phrenic motor responses and entrain breathing
Yang et al. (2019) [66]	Rats	LGMedSupply	C4–T2	200	330	Up to 1.5 mA	Cortical stimulation	75% of motor threshold for evoked response
Samejima et al. (2021) [68]	Rats	Custom	C6	400	50–100	300–1000μA	BCI	Individually set to invoke elbow extension
Barra et al. (2022) [19]	*Macaca fascicularis*	Custom	C6–T1	200–400	20–120	600–1400μA	Cortical stimulation	Stimulation triggered in phase with voluntary movement; individualized optimization
Pal et al. (2022) [71]	Rats	Custom	C5–C6	200	5	N.R.	Cortical stimulation	M1 stimulation at 110% of the threshold for evoking a cortical MEP followed 10 ms later by spinal stimulation at 90% of the threshold for generating a spinal MEP
Transcutaneous stimulation								
Song et al. (2016) [67]	Rats	StimTentCom	C4–T2; M1 region	200	100; 0.2	100–400/ 50–100μA	Cortical stimulation	Paired M1 (0.2 Hz) and spinal stimulation (100 Hz, 5 pulses/burst) with 10 ms interstimulus interval
Murray et al. (2017) [26]	Human	UniPatch	C5–T2	1000	0.2	Up to 150 mA	TMS	Intensities increased from sub-threshold to suprathreshold
Zareen et al. (2017) [25]	Rats	StimTentCom	C4–T2; M1 region	200	330; 250	Up to 1.5 mA	Cortical stimulation	10 biphasic pulses at 250 Hz every 2 s; amplitude set at 75% of motor threshold
Inanici et al. (2018) [49]	Human	Axelgaard	C3–C4, C6–C7	1000	30 (10)	80–120 mA	–	Individually titrated to comfort
Gad et al. (2018) [59]	Human	Axelgaard	C3–C4, C6–C7	1000	30 (10)	10–250 mA	–	Adjusted to enable maximal grip strength without causing discomfort
Benavides et al. (2020) [52]	Human	Axelgaard	C5–C6	200	30 (5)	79.2±12.2 mA	TMS	Intensity set to evoke biceps root-evoked potential ≥50μV in 5/10 trials
Zhang et al. (2020) [51]	Human	STIMEX	C3–C4, C7–T1	1000	30 (10)	Up to 80 mA	–	Adjusted to enable maximal grip strength without causing discomfort
Gad et al. (2020) [62]	Human	TESCoN	C5–C6	1000	30 (10)	20 mA	–	Adjusted for dose–response curves of inspiratory capacities and forced expiratory volume
Tefertiller et al. (2021) [57]	Human	Axelgaard	C3–C4, C6–C7, T11–T12	1000	30 (10)	45–150 mA	–	Adjusted until optimal functional movement of the targeted area was achieved in a joint below the level of injury
Inanici et al. (2021) [48]	Human	Axelgaard	C3–C4, C6–C7	1000	30 (10)	80–120 mA	–	Individually titrated to comfort
Huang et al. (2022) [61]	Human	Axelgaard	C4–C5	1000	30	40–180 mA	–	Individually titrated to elicit maximal handgrip; stimulation timed concurrently to voluntary handgrip attempt
McGeady et al. (2022) [54]	Human	Axelgaard	C4–C6	1000	30	40–55 mA	Motor imagery	Individually titrated to comfort
Zhang et al. (2023) [50]	Human	STIMEX	C3–C5	1000	30	Up to 120 mA	–	Individually adjusted for subthreshold upper extremity muscle activation
Oh et al. (2023) [70]	Human	Axelgaard	C6–T1	500	30	20–70 mA	–	Adjusted for maximum hand grip
García-Alén et al. (2023) [55]	Human	Axion	C3–C4, C6–C7	1000	30 (10)	45–86 mA	Robotic exoskeleton	90% of RMT induced by single-pulse tSCS at the abductor pollicis brevis
Chandrasekaran et al. (2023) [60]	Human	Custom	C4–T1	500	50	140–160 mA	–	Adjusted for maximal tolerable intensity during ABT
Moritz et al. (2024) [56]	Human	LIFT (ARCex)	C3–C7	1000	30 (10)	10–180 mA	–	Individually titrated to comfort; electrode placement individualized based on desired effect
Singh et al. (2024) [63]	Human (Children)	Syrtenty	C3–C4, C6–C7, T10–T11	1000	30 (10)	20–70 mA	–	Adjusted to enable maximal hand-grip force at subthreshold intensity
Verma et al. (2025) [58]	Human	Anuevo	C3–T1	500	30	<90 mA	–	Maximum tolerable intensity just below motor threshold
Capozio et al. (2025) [53]	Human	Axelgaard	Above/below lesion	1000	30	40–70 mA	TMS	Individually titrated to 80–90% of RMT
Capozio et al. (2025) [69]	Human	Axelgaard	C5–C6 midline	1000	30 (5)	30–60 mA	TMS, motor imagery	Individually titrated to 80–90% of RMT

Abbreviations: RMT = Resting Motor Threshold; BCI = brain–computer interface; TMS = Transcranial Magnetic Stimulation; ABT = Activity-Based Therapy; N.R. = Not Reported.

**Table 3:** Summary of functional outcomes in human studies.

Authors	GRASSP	ISNCSCI	Dyn.	Main Finding
Epidural stimulation				
Lu et al. (2016) [18]	–	↑	↑	Immediate and session-to-session motor gains in 2/2 participants; three-fold increase in hand strength with stimulation; upper-extremity motor score increased by 23 and 16 points; improved self-care and mobility.
Transcutaneous stimulation				
Murray et al. (2017) [26]	–	–	–	Reduced spasticity and spasms, reversal of anhidrosis.
Inanici et al. (2018) [49]	↑	↑	↑	Improvements of 52 points on GRASSP, 14 points on UEMS, 2- to 7-fold increase in pinch strength in left and right hand, respectively; overall sensation and neurological level of injury improved from C3 to C4. Functional gains persisted for over 3 months after stimulation.
Gad et al. (2018) [59]	–	–	↑	Grip strength increased by 325% with stimulation and 225% off stimulation after 8 sessions.
Benavides et al. (2020) [52]	↑	–	–	Immediate GRASSP improvements observed in a single session.
Zhang et al. (2020) [51]	↑	–	↑	GRASSP improved by 24 points and handgrip strength increased (left: 283%, right: 30%); gains sustained for 3 months.
Gad et al. (2020) [62]	–	–	–	Improved breathing and cough effectiveness; benefits persisted beyond the stimulation period.
Tefertiller et al. (2021) [57]	–	↑	–	AIS grade improved in 2/7 participants (B to C; C to D); sensation increased in 5/7; all 4/4 AIS A participants were able to activate lower extremities with stimulation.
Inanici et al. (2021) [48]	↑	↑	↑	GRASSP scores increased; UEMS improved by up to 8 points. AIS grade improved in 1/6 participants (AIS C to D); all 6/6 maintained gains for at least 3–6 months beyond stimulation. Additional improvements in bladder function, thermoregulation, heart-rate stability, and quality of life.
Huang et al. (2022) [61]	–	–	↑	Grip force increased in 5/10 participants with measurable baseline force.
McGeady et al. (2022) [54]	↑	↑	↑	GRASSP increased by 35 points with associated strength gains and improved neurological level of injury; brain–computer interface priming enhanced gains.
Zhang et al. (2023) [50]	↑	–	↑	Five-fold improvement in GRASSP in AIS A; GRASSP increased by 10–33 points; up to three-fold increase in upper-extremity strength; gains maintained for 1 month in all 4/4 participants.
Oh et al. (2023) [70]	–	–	↑	Grip strength increased only with combined stimulation and training.
García-Alén et al. (2023) [55]	↑	↑	↑	GRASSP strength and pinch improved; also increases in SCIM scores and quality of life.
Chandrasekaran et al. (2023) [60]	–	↑	↑	Up to 1136% increase in force; tactile sensation improved.
Moritz et al. (2024) [56]	↑	↑	↑	Seventy-two percent of participants exceeded MCID; pinch, grip, UEMS, and SCIM scores increased; autonomic function (bladder and blood-pressure regulation) improved in some; significant improvements in patient-reported quality of life.
Singh et al. (2024) [63]	–	–	↑	Handgrip increased in 6/7 participants; more than 20% force increase in 3 participants; safety and tolerability in children confirmed.
Verma et al. (2025) [58]	–	–	↑	Grip strength increased; minimal residual force at baseline predicted response.
Capozio et al. (2025) [53]	↑	–	–	GRASSP improved in 4/5 participants; sensation improved in 3/5.
Capozio et al. (2025) [69]	–	–	–	Dexterity increased after a single session, with or without stimulation.

Abbreviations: GRASSP = Graded and Redefined Assessment of Strength, Sensibility, and Prehension; ISNCSCI = International Standards for Neurological Classification of Spinal Cord Injury; Dyn. = dynamometry; MCID = Minimal Clinically Important Difference; UEMS = Upper Extremity Motor Score; SCIM = Spinal Cord Independence Measure; AIS = ASIA Impairment Scale.

## 4. Discussion

The aim of this study was to obtain a validated overview of the current evidence of cervical SCS for functional rehabilitation after SCI, with a focus on motor, sensory, respiratory, and autonomic outcomes, and to evaluate the methodological quality of the available studies. To this end, we conducted a systematic literature review, including human and animal studies, with accompanying risk-of-bias assessment for human studies. Across the included human studies, cervical SCS was generally well tolerated within the limitations of small, largely uncontrolled studies with no major stimulation-related adverse events recorded. Minor side effects, such as transient discomfort, were infrequent and consistent with the established safety profile of SCS in other neurological contexts [10,72].

Preclinical studies offer important mechanistic insights. They demonstrate that cervical stimulation can activate forelimb motor pools, strengthen spared corticospinal connections, and facilitate long-term improvements in skilled motor function [17,27,30,71,73]. Notably, some models demonstrate that stimulation can induce lasting plastic changes rather than only transient facilitation. McPherson et al. showed that activity-dependent stimulation produced durable recovery of reaching ability accompanied by strengthening of spared corticospinal projections, while Zareen et al. demonstrated axonal outgrowth of corticospinal fibers when spinal stimulation was paired with cortical neuromodulation [17,25]. These data suggest that cervical SCS may act not just as an assistive technology but as a therapeutic intervention promoting structural reorganization. Additional work highlights its ability to engage respiratory-related circuits, reinforcing relevance for patients with high cervical injuries [37,62,74,75]. The emerging body of human research reinforces and extends the preclinical evidence base. Across 20 included clinical studies, cervical SCS has been shown to have preliminary assistive and therapeutic effects on upper limb motor function, with occasional reports of enhanced respiratory capacity. However, most investigations were uncontrolled pilot trials or case reports, leaving the certainty of evidence low. Only Huang et al., a sham-controlled crossover trial in 10 participants, and Moritz et al., a multicenter prospective study with 60 participants, were judged to be at a moderate risk of bias [56,61]. These provide early but important steps toward rigorous evaluation. Importantly, aside from the trial by Lu et al., epidural cervical SCS studies in SCI remain limited to animal studies, with no larger clinical series demonstrating sustained functional outcomes [18]. By contrast, epidural approaches at the cervical level have been explored more recently in poststroke hemiparesis, though these still required laminectomy and open surgical implantation, whereas SCI research has largely pivoted toward non-invasive transcutaneous stimulation [20,22]. This shift likely reflects the technical and safety challenges associated with cervical epidural lead implantation in the setting of SCI, including altered anatomy, scarring, and concerns regarding surgical risk in a neurologically vulnerable region. In addition to stimulation alone, several studies integrated adjunctive interventions such as motor cortex stimulation, brain–computer interface priming, motor imagery, robotic exoskeleton training, and task-specific activity-based practice. Although heterogeneous in design, these approaches consistently reported greater or more durable functional improvements than stimulation alone. This supports the hypothesis that cervical SCS works best not as a stand-alone restorative therapy but rather as a facilitator of neuroplasticity, amplifying the effects of motor practice and supraspinal input. These findings corroborate reports on lumbosacral SCS, where task-specific training has been effective in translating the acute effects of stimulation into lasting functional gains [11,12,76]. Future clinical trials should therefore prioritize protocols that systematically pair stimulation with rehabilitation or other neuromodulation methods, as this combined approach is most likely to yield meaningful and durable recovery. A recurring clinical question concerns the degree of residual function required to benefit from cervical SCS. The studies included in this review encompassed both motor-complete (AIS A–B) and motor-incomplete (AIS C–D) injuries. Improvements in hand strength and dexterity were reported even in some participants classified as AIS A; however, these were often single cases and typically involved at least minimal residual motor output detectable on EMG or dynamometry. Larger series and controlled studies suggest that individuals with preserved voluntary activation, even if weak (AIS B–D), are more likely to experience robust gains. For example, Huang et al. and Verma et al. showed that minimal baseline handgrip force was a prerequisite for substantial strength improvements [58,61]. Current evidence therefore indicates that cervical SCS can modulate function across a spectrum of injury severities, but that the predictability and magnitude of response are greatest when some descending drive is preserved. More systematic, stratified trials are needed to define clear thresholds of residual function and to determine whether truly motor-complete injuries without any measurable output can benefit to a similar extent.

Important limitations remain. The current literature is dominated by small sample sizes, heterogeneous injury profiles, variable stimulation protocols, and unblinded outcome assessments. These factors limit generalizability and make it difficult to identify which patients are most likely to benefit. Although motor outcomes in the human studies were assessed using validated measures, substantial variability in the choice of assessments and the frequent use of adjunctive interventions limited comparability across studies and precluded a formal meta-analysis. Few studies incorporated patient-reported outcomes or long-term follow-up, leaving gaps in understanding durability and real-world impact [77].

## 5. Conclusions

In summary, this systematic review addresses its stated objectives by synthesizing available preclinical and clinical evidence on cervical spinal cord stimulation for functional rehabilitation following SCI, identifying preliminary assistive and therapeutic effects, and evaluating the methodological limitations that currently constrain the strength of this evidence. Based on the reviewed evidence, several priorities for future research emerge. First, larger sham-controlled and blinded clinical trials are essential to establish efficacy and to separate stimulation-specific effects from placebo or training-related gains. To overcome this challenge, future studies may consider alternative electrode placements that preserve cutaneous sensation without engaging spinal circuits or the use of active comparator conditions rather than inert shams. Second, optimization, personalization, and safety of stimulation parameters should be pursued through systematic exploration of electrode placement, optimal stimulation configurations, and activity-dependent paradigms. Third, integration with adjunctive rehabilitation strategies appears critical, as stimulation likely acts as a facilitator of neuroplasticity rather than a stand-alone intervention. Finally, identifying which patient subgroups are most likely to benefit, including potential age- and sex-related differences in functional responsiveness, will be critical for guiding clinical decision-making and resource allocation. Given the profound impact that even modest improvements in hand or respiratory function can have for people with tetraplegia, the continued systematic development and rigorous evaluation of cervical SCS remains a clear research priority.

## Data Availability

The raw data supporting the conclusions of this article will be made available by the authors on request.

## Abbreviations

The following abbreviations are used in this manuscript:

AIS	ASIA Impairment Scale
ASIA	American Spinal Injury Association
BCI	Brain–Computer Interface
CI	Confidence Interval
CNS	Central Nervous System
EES	Epidural Electrical Stimulation
EMG	Electromyography
GRASSP	Graded and Redefined Assessment of Strength, Sensibility, and Prehension
ISMS	Intraspinal Microstimulation
ISNCSCI	International Standards for Neurological Classification of Spinal Cord Injury
MCID	Minimal Clinically Important Difference
MEP	Motor Evoked Potential
MRI	Magnetic Resonance Imaging
N.A.	Not Applicable
N.R.	Not Reported
PRISMA	Preferred Reporting Items for Systematic Reviews and Meta-Analyses
PRO	Patient-Reported Outcome
PROSPERO	International Prospective Register of Systematic Reviews
RCT	Randomized Controlled Trial
RMT	Resting Motor Threshold
SCAP	Spinal Cord Associative Plasticity
SCI	Spinal Cord Injury
SCIM	Spinal Cord Independence Measure
SCIM-III	Spinal Cord Independence Measure, Version III
SCS	Spinal Cord Stimulation
sMEP	Surface Motor Evoked Potential
tSCS	Transcutaneous Spinal Cord Stimulation
TMS	Transcranial Magnetic Stimulation
UEMS	Upper Extremity Motor Score
QoL	Quality of Life

## Appendix A

**Table A1:** Risk of bias assessment for all included human studies.

Study (Year)	Study Design	Confounding (Patient Selection, Injury Severity)	Intervention Classification & Reporting	Deviations from Intended Intervention	Missing Data/Attrition	Outcome Measurement (Blinding/Objectivity)	Selective Reporting	Overall Risk of Bias
Lu et al. (2016) [18]	Case series (cervical epidural stimulation in chronic SCI)	Serious—very small sample size (n = 2), no control group, variability in chronicity	Low–intervention (epidural electrode placement, stimulation parameters) clearly described	Low–stimulation applied as intended, intraoperative testing documented	Moderate—limited reporting on attrition; unclear if any patients excluded post-implantation	Serious—outcomes (hand strength, EMG, volitional control) assessed without blinding; risk of expectation bias	Moderate—selective emphasis on functional improvements, limited discussion of null or adverse findings	Serious
Murray et al. (2017) [26]	Case study (cervicothoracic tSCS in SCI, n = 1)	Critical—single participant, exploratory design, no control group	Low—stimulation protocol (site, frequency, sessions) described in detail	Low—intervention delivered as intended	Low—no attrition (single case)	Serious—outcomes (EEG, MEPs, motor activity) assessed without blinding; subjective interpretation risk	Moderate—selective focus on neurophysiological improvements, limited discussion of null or negative findings	Serious–Critical
Inanici et al. (2018) [49]	Case study (cervical tSCS in chronic tetraplegia, n = 1)	Critical—single participant, no control group	Low—stimulation parameters (site, intensity, frequency) well described	Low—intervention delivered as intended	Low—no attrition (single case)	Serious—motor and functional outcomes assessed without blinding; potential observer bias	Moderate—focus on positive motor improvements, limited reporting of variability or negative results	Serious–Critical
Gad et al. (2018) [59]	Case series (cervical tSCS in SCI, n = 6)	Serious—small heterogeneous cohort, varied chronicity and injury severity, no control group	Low—stimulation protocol (site, electrode configuration, parameters) clearly described	Low—intervention delivered as intended across sessions	Moderate—some incomplete follow-up and variability in session numbers	Serious—outcomes (hand strength, EMG, functional tasks) assessed without blinding; observer bias possible	Moderate—emphasis on positive motor outcomes, limited discussion of non-responders	Serious
Benavides et al. (2020) [52]	Case series (cervical tSCS in chronic tetraparesis, n = 10)	Serious—small cohort, varied injury severity and chronicity, no control group within SCI sample	Low—stimulation protocol (electrode placement, frequency, parameters) clearly described	Low—intervention delivered as intended	Moderate—follow-up limited to short-term sessions; attrition not clearly detailed	Serious—outcomes (TMS, MEPs, motor responses) assessed without blinding; potential interpretive bias	Moderate—selective emphasis on corticospinal excitability changes, limited reporting on individual variability	Serious
Zhang et al. (2020) [51]	Case study (cervical tSCS in complete tetraplegia, n = 1)	Critical—single participant, no comparator, no ability to control for confounding	Low—intervention (electrode placement, frequency, intensity) well described	Low—stimulation delivered as intended	Low—no attrition (single case)	Serious—outcomes (upper extremity strength, hand function tests) assessed without blinding; observer and reporting bias likely	Moderate—emphasis on positive motor outcomes, limited acknowledgment of limitations of single case	Serious–Critical
Gad et al. (2020) [62]	Case study (cervical tSCS for respiratory control in chronic tetraplegia, n = 1)	Critical—single participant, no control group, findings not generalizable	Low—intervention (electrode placement, stimulation parameters) well described	Low—intervention delivered as intended	Low—no attrition (single case)	Serious—respiratory outcomes (ventilation, EMG) assessed without blinding; observer bias possible	Moderate—emphasis on positive physiological responses, limited reporting of variability or potential null effects	Serious–Critical
Tefertiller et al. (2021) [57]	Case series (tSCS in chronic SCI, n = 7)	Serious—small heterogeneous cohort with varied injury levels and severities, no control group	Low—stimulation protocol (electrode placement, parameters, training) well described	Low—intervention delivered as intended	Moderate—limited follow-up, incomplete reporting of attrition	Serious—outcomes (motor function, grip, task performance) assessed without blinding; potential observer bias	Moderate—selective emphasis on functional improvements, limited reporting of negative or null results	Serious
Inanici et al. (2021) [48]	Prospective cohort study (cervical tSCS in chronic SCI, n = 6)	Serious—small cohort, varied AIS grades and chronicity, no control group	Low—intervention (tSCS protocol, electrode placement, parameters) clearly described	Low—intervention delivered as intended	Low—all participants completed planned sessions, minimal attrition	Serious—motor and functional outcomes assessed without blinding; possible observer and participant bias	Low to Moderate—outcomes comprehensively reported, but limited negative data presented	Serious
Huang et al. (2022) [61]	Randomized, sham-controlled crossover trial (cervical tSCS in severe SCI, n = 10)	Serious—small cohort, heterogeneous injury severity, limited generalizability despite crossover design	Low—stimulation parameters (site, intensity, frequency) clearly reported	Low—intervention delivered as intended	Low—all participants completed protocol, no attrition	Moderate—motor outcomes assessed without full blinding of assessors; participants blinded to sham vs. active	Low—outcomes comprehensively reported, including individual data	Moderate
McGeady et al. (2022) [54]	Case study (BCI priming + cervical tSCS in chronic SCI, n = 1)	Critical—single participant, exploratory design, no control or comparator	Low—intervention (BCI priming protocol and tSCS parameters) described in detail	Low—intervention delivered as intended	Low—no attrition (single case)	Serious—motor and functional outcomes assessed without blinding; high risk of expectation and observer bias	Moderate—focus on feasibility and positive effects, limited discussion of null findings or generalizability	Serious–Critical
Zhang et al. (2023) [50]	Conference abstract (tSCS + activity-based training in cervical SCI, n = 4)	Critical—very small cohort, conference abstract format, limited methodological detail, no control group	Serious—intervention parameters only briefly described; limited reproducibility	Moderate—intervention likely delivered as intended but insufficient detail to confirm	Serious—attrition and follow-up not reported	Serious—outcomes reported in summary only; no blinding; high reporting bias risk	Critical—selective emphasis on feasibility and positive findings; lack of detailed data	Serious–Critical
Oh et al. (2023) [70]	Case series (cervical tSCS + task-specific training in chronic SCI, n = 4)	Serious—very small sample, heterogeneous injuries, no control group	Low—stimulation parameters (electrode placement, frequency, intensity) and training protocol described in detail	Low—intervention delivered as intended	Moderate—follow-up limited, attrition reporting incomplete	Serious—motor outcomes (hand strength, task performance) assessed without blinding; observer and participant bias possible	Moderate—focus on positive findings, limited reporting of non-responders or null results	Serious
García-Alén et al. (2023) [55]	Clinical trial (cervical tSCS + robotic exoskeleton rehabilitation, n = 15)	Serious—modest sample size, heterogeneous chronic cervical injuries, limited randomization details, no blinded control	Low—intervention protocols (tSCS parameters, exoskeleton training) described clearly	Low—interventions delivered as intended	Moderate—short-term intervention; attrition not fully described	Serious—outcomes (motor scores, functional tasks) assessed without blinding; potential observer bias	Moderate—results emphasize improvements; limited discussion of non-responders or null effects	Serious
Chandrasekaran et al. (2023) [78]	Pilot study (targeted cervical tSCS in chronic SCI, n = 7)	Serious—small cohort, mixed injury severities, no control group	Low—stimulation protocol (targeted electrode placement, parameters) described in detail	Low—intervention delivered as intended	Low—all participants completed intervention, minimal attrition	Serious—outcomes (strength, tactile sensation, functional tasks) assessed without blinding; observer and participant bias possible	Moderate—selective emphasis on improvements, limited detail on negative results or variability	Serious
Moritz et al. (2024) [56]	Multicenter prospective clinical trial (cervical tSCS in chronic tetraparesis, n = 60+)	Moderate—relatively large sample, but no sham-control group; heterogeneous injury levels and chronicity	Low—intervention (tSCS protocol, electrode placement, stimulation parameters, training regimen) described in detail	Low—intervention delivered as intended across sites with standardized procedures	Low—minimal attrition reported; safety and adherence tracked	Moderate—outcomes (grip strength, hand function, SCIM, PROs) not blinded; potential performance and observer bias	Low—outcomes comprehensively reported per protocol, including adverse events and non-responders	Moderate
Singh et al. (2024) [63]	Prospective feasibility study (cervical and thoracic tSCS in pediatric chronic SCI, n = 6)	Serious—very small sample, pediatric population with variable injury levels and chronicity, no control group	Low—intervention (cervical/thoracic electrode placement, stimulation parameters, safety monitoring) clearly described	Low—intervention delivered as intended	Low—no attrition reported during short-term intervention	Serious—motor function outcomes assessed without blinding; potential observer bias	Moderate—emphasis on feasibility and safety; limited discussion of negative or null results	Serious
Verma et al. (2025) [58]	Prospective case series (cervical tSCS in chronic SCI, n = 5)	Serious—very small cohort, varied injury severities and chronicity, no control group within SCI	Low—stimulation parameters (cervical electrode placement, frequency, intensity) well described	Low—intervention delivered as intended	Low—all participants completed protocol, no attrition	Serious—outcomes (motoneuron firing via EMG, hand motor function) assessed without blinding; risk of observer bias	Moderate—focus on facilitation and motor improvements, limited discussion of non-responders or null findings	Serious
Capozio et al. (2025a) [53]	Preprint (bimanual task practice + cervical tSCS in chronic SCI, n = 5)	Critical—preprint (not peer-reviewed), very small sample, no control group, heterogeneous injuries	Serious—intervention parameters described but some methodological details limited in preprint format	Low—intervention delivered as intended	Moderate—short-term follow-up; attrition reporting incomplete	Serious—outcomes (hand motor function, spinal plasticity measures) assessed without blinding; risk of observer bias	Serious—emphasis on positive findings, limited reporting of null or negative outcomes; preprint format limits transparency	Serious–Critical
Capozio et al. (2025b) [69]	Single-session experimental study (motor imagery + cervical tSCS in chronic SCI, n = 8)	Serious—very small sample, heterogeneous injuries, no control/sham condition	Low—stimulation protocol and motor imagery task clearly described	Low—intervention delivered as intended in all participants	Low—no attrition (single-session study)	Serious—outcomes (manual dexterity, cortical excitability via TMS) assessed without blinding; potential measurement bias	Moderate—focus on positive acute effects, limited reporting of variability or null findings	Serious
